# Toxic Element Contamination of Natural Health Products and Pharmaceutical Preparations

**DOI:** 10.1371/journal.pone.0049676

**Published:** 2012-11-21

**Authors:** Stephen J. Genuis, Gerry Schwalfenberg, Anna-Kristen J. Siy, Ilya Rodushkin

**Affiliations:** 1 Faculty of Medicine, University of Alberta, Edmonton, Alberta, Canada; 2 Department of Geosciences and Environmental Engineering, Luleå University of Technology, Luleå, Sweden; 3 ALS Scandinavia AB, Luleå, Sweden; Concordia University Wisconsin, United States of America

## Abstract

**Background:**

Concern has recently emerged regarding the safety of natural health products (NHPs)–therapies that are increasingly recommended by various health providers, including conventional physicians. Recognizing that most individuals in the Western world now consume vitamins and many take herbal agents, this study endeavored to determine levels of toxic element contamination within a range of NHPs.

**Methods:**

Toxic element testing was performed on 121 NHPs (including Ayurvedic, traditional Chinese, and various marine-source products) as well as 49 routinely prescribed pharmaceutical preparations. Testing was also performed on several batches of one prenatal supplement, with multiple samples tested within each batch. Results were compared to existing toxicant regulatory limits.

**Results:**

Toxic element contamination was found in many supplements and pharmaceuticals; levels exceeding established limits were only found in a small percentage of the NHPs tested and none of the drugs tested. Some NHPs demonstrated contamination levels above preferred daily endpoints for mercury, cadmium, lead, arsenic or aluminum. NHPs manufactured in China generally had higher levels of mercury and aluminum.

**Conclusions:**

Exposure to toxic elements is occurring regularly as a result of some contaminated NHPs. Best practices for quality control–developed and implemented by the NHP industry with government oversight–is recommended to guard the safety of unsuspecting consumers.

## Introduction

The issue of harm related to healthcare provision has become a persistent problem that has been shrouded in silence. [1], [2] Most people in the Western world believe there is adequate protection when they or their loved ones receive health advice or intervention. [2] Yet, considerable data from varying locales and demographics paints a different story. [3]–[19] Rather than rare occurrences, adverse events related to provision of health services are common; they frequently cause serious harm and most are entirely preventable. [2]–[18] An emerging public health concern relates to hazards posed by exposure to toxicants [20], [21] through contaminated everyday merchandise, [22] including natural health products (NHPs). The contemporary reality is that most individuals in the Western world now consume some form of NHP [23] and many of these products are increasingly recommended by health providers – a recent survey found about 38% of Canadian physicians now recommend some NHPs to their patients. [24].

Accordingly, this study was designed to determine if toxic element contamination of NHPs is a routine occurrence or a sporadic event. A variety of common pharmaceutical preparations were tested for comparison purposes.

### Background

Various items including foods, toys, cosmetics and other personal care products have recently been found to contain toxic compounds[25]–[28] including lead, arsenic, mercury, cadmium as well as an array of synthetic agents – raising concern about contamination in common items used by much of the population. NHPs include vitamins, herbal products, probiotics, homeopathic medications and various supplements containing nutrients or other compounds purported to benefit health. The number of assorted dietary supplements has risen to around 55,000 in the United States, [29] with an estimated 60% of Americans now using NHPs. [23] 50% of Europeans on average consume NHPs, [30], [31] and in Canada, approximately 71% of the population uses NHPs, with 38% doing so on a daily basis. [32] Vitamins are the most commonly consumed product – used by 57% of Canadians, followed by 15% using Echinacea and 11% using other herbal, fungal or algal products. [32].

**Figure 1:**
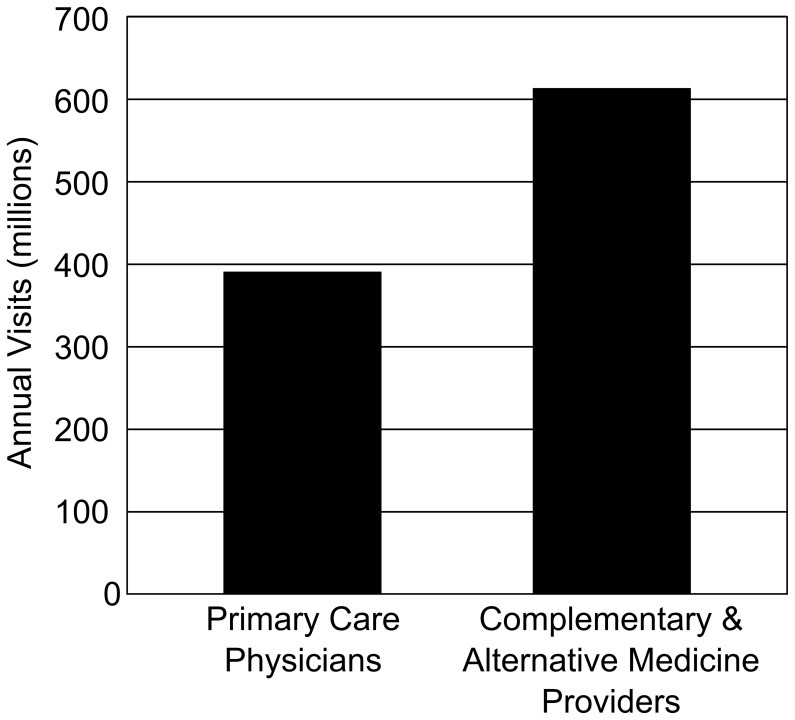
Use of Alternative medicine in relation to Conventional Medicine [**34**].

Many health providers now recommend NHPs including prenatal vitamins, iron supplementation, calcium, and vitamin D for a range of recognized indications including deficiency states such as rickets and anemia, as well as illnesses such as multiple sclerosis. [33] Many consumers are also pursuing natural and holistic approaches to medicine (Figure 1) [34] with the result that NHPs have found a ready market in non-allopathic medicine. Over the past two decades, use of alternative medicine has increased, [34], [35] with an annual estimated $700 million spent on all products and therapies in England, [31] $7.84 billion in Canada, [36] and $33.9 billion in the USA with $14.8 billion spent specifically on NHPs. [37].

With increases in globalization, cultural remedies from Chinese, Ayurvedic, and other traditions have become more available to international consumers, offering unfamiliar products with unfamiliar adverse effects. Thus, beyond questions of efficacy and drug interactions, the inherent safety of NHPs has come under increasing scrutiny in the public health community. [35], [38], [39] Consumers are similarly eager for information, with 84% of Canadians believing that “more needs to be done to inform Canadians about the safe use of NHPs”. [32] Although conventional pharmaceuticals are by no means innocuous, [2], [40], [41] international research indicates that NHPs are not always completely safe either. Contamination with toxicants including lead, mercury, arsenic, and other toxic elements has been documented in a variety of NHPs from various parts of the globe, particularly some parts of Asia and the Orient. [42].

### Ayurveda

Ayurvedic practices stem from the Vedic culture of southern Asia, and date back over 5000 years. Rather than a purely structural, organ-based approach to health, Ayurveda focuses on the functions of organ systems and the body as a whole. [43] Key to the concept of health is the unique energy patterns of each individual, reflecting a combination of the three energies: *vata* (metabolism), *pitta* (structure, stability) and *kapha* (movement). All clinical symptoms are assessed as an imbalance between these energies; restoration of balance often involves changes in lifestyle and diet, habits of meditation and mindfulness, detoxification, [44] as well as various herbal preparations. [45].

Some Ayurvedic preparations have been found to contain significant amounts of lead, mercury and arsenic. [46], [47] It is sometimes thought within Ayurvedic tradition that metals and metalloids should be included with minerals to maintain a proper balance for health. Thus, metal content in Ayurvedic supplements may result from intentional additives (that have undergone traditional cleansing procedures), rather than from contamination. [48], [49] Examples of these purifying procedures have been documented, [50], [51] but convincing evidence is lacking to support the efficacy of these procedures in decreasing the toxicity of harmful substances present in the final preparations. [49] Toxins leaching from contaminated soil may also contribute to the toxicant content of the raw materials. [52].

Ayurvedic supplements containing toxic elements are widely available in the United States. [46], [53] Lead exposure has been associated with episodes of neurological damage following Ayurvedic NHP consumption, especially in pediatric populations. Status epilepticus, congenital sensorineural deafness, infant encephalopathy, [54] and developmental delays have all been reported after use of lead-contaminated Ayurvedic NHPs. [46] Acute presentations also include GI symptoms, [55], [56] hepatotoxicity [57], [58] and hematopoietic toxicity. [56], [59] Effects of lead in Ayurvedic preparations may also lead to subacute presentations, with toxic blood levels noted for more than 30 days in some patients after one-time consumption. [60].

Other toxicant related problems have resulted from consumption of Ayurvedic preparations. Mercury from Ayurvedic NHPs has been associated with weight loss, diarrhea, sweating, tremors, paresthesias and peripheral neuropathy, [61] as well as skin lesions in topical preparations. [62] A case of chronic arsenic toxicity secondary to Ayurvedic medications presented with skin lesions (punctuate palmoplantar keratoderma and leucomelanoderma) and portal hypertension. [63] In review, toxic element contamination of Ayurvedic NHPs is a well-established concern.

### Traditional Chinese Medicine (TCM)

Dating back thousands of years, traditional Chinese Medicine (TCM), like Ayurveda, arises from a philosophy of balance as well as pattern-based diagnosis and treatment. Herbs may be classified according to taste (sour, bitter, sweet, pungent, and salty), ‘temperature’ (cold, warm, hot, cool) or direction (ascending, descending, floating, and sinking). Symptoms of illness are categorized, then treated with opposing herbs. [35].

Lead, [60] mercury, arsenic, copper, cadmium, and thallium have been reported in TCM products purchased in the United States and China,[64]–[69] intended to treat issues ranging from gingivitis and sore throats to appendicitis and coronary disease. [65] Research from Singapore, where TCM supplements are tightly controlled, showed heavy metal contaminants in 138 of 3320 products screened from 1990–2001. [70] Of the contaminated products, mercury was found in 51.4%, arsenic in 34.8%, lead in 14.5% and copper in 0.7%. [70].

**Table 1:** Established Toxicant Limits in Supplements (mcg/day).

Toxic Element	U.S. California Proposition 65,^[85]^ and EnvironmentalProtection Agency ^[102]^	EuropeanUnion^[103], [104], [105]^	Australia ^[106]^	World HealthOrganization ^[85], [103]^	Gestational Limits ^[107], [108]^
Mercury (Hg)	2	4.2	2.4 Inorganic Hg0.96 Methyl Hg	1.37 (Methyl Hg inchildren)	O.6 for Methyl Hg
Lead	15	21	NE	21	Concern at low levels. No level yet established as acceptable
Cadmium	4.1	6	15	6	NE
Arsenic	10	13.0	NE	12.85	NE
Aluminum	7,000	4,286	12,000	NE	NE
Barium	1,200	NE	NE	NE	NE
Antimony	2.8	36	NE	NE	NE
Thallium	70	NE	NE	NE	NE
Tin	200	NE	NE	NE	NE
Cesium	NE	NE	NE	NE	NE

NE – Not established.

European/WHO/Australian levels were established by convention as representing 10% of the daily total toxicant intake after conversion of values expressed in mg/kg/week for an average adult weight of 60 kg.

**Table 2:** Overall Results of Toxic Element Contamination***.

Element in mcg	Mercury	Lead	Cadmium	Arsenic	Aluminum	Barium	Antimony	Thallium	Tin	Cesium
Allowable limit/day* (micrograms)	2	15	4.1	10	7,000	1,400	2.8	70	200	NE
**Natural Health Products (NHPs) – Overall**										
(N) tested	121	121	121	100	121	121	72	65	65	65
Average daily exposure (mean)	0.366	1.49	0.199	21.7	573	59.3	0.126	0.0384	0.608	0.167
Standard Deviation	3.80	5.33	0.803	202	1,590	138	0.372	0.0803	1.88	0.400
Highest daily exposure in single sample	41.8	51.4	6.81	2,020	12,900	894	2.32	0.354	13.2	2.34
Average annual exposure	134	545	72.9	7,910	209,000	21,700	45.9	14.0	222	61.0
Number exceeding daily limit	1	2	2	5	2	0	0	0	0	N/A
Percent with detectable contaminant*	31.4	51.2	33.1	57	82.6	81.8	37.5	64.6	67.7	66.1
**Pharmaceuticals – Overall (n = 49)**										
Average daily exposure (mean)	0.0007	0.0237	0.0035	0.0069	336	0.200	0.012	0	0.024	0.0026
Standard Deviation	0.0007	0.033	0.0098	0.01	104	0.405	0.035	0	0.042	0.103
Highest daily exposure in single sample	0.0023	0.147	0.0241	0.0461	381	1.93	0.072	0.00	0.117	0.0694
Average annual exposure	0.256	8.66	1.28	2.52	123,000	73.2	4.38	0	8.77	0.950
Number exceeding daily limit	0	0	0	0	0	0	0	0	0	N/A
Percent with detectable contaminant*	91.8	89.8	89.8	93.8	100	100	91.8	0	100	89.8
**North American NHPs excluding Chinese, Ayurvedic and marine products**										
(N) tested	91	91	91	72	91	91	49	44	44	44
Average daily exposure (mean)	0.0146	0.362	0.0918	0.782	160	41.3	0.0853	0.0094	0.090	0.0411
Standard Deviation	0.0781	1.01	0.334	3.16	337	123	0.340	0.0122	0.165	0.112
Highest daily exposure in single sample	0.714	6.54	1.86	23.9	2,000	894	2.32	0.039	0.40	0.683
Average annual exposure	5.33	132	33.5	286	58,600	15,100	31.2	3.43	32.9	15.0
Number exceeding daily limit	0	0	1	2	0	0	0	0	0	0
Percent with detectable contaminant**	25.3	39.6	25.3	51.4	71	69.2	32.6	61.4	54.5	61.4

Category of NHP indicates classification of product in store or company where purchased. This does not necessarily indicate where source materials for the NHPs are initially manufactured or derived.

* Limits from U.S. California Proposition ^65,^ ^[85]^ and Environmental Protection Agency ^[102]^ as per Table 2.

** The limit of detection will vary between analytical laboratories and may thus influence the percent with detectable contaminants when levels are at low concentrations.

*** Tables 3–6 should be interpreted together and in context as there were single outliers (such as the Hg level in one Chinese NHP) that radically skewed the mean and standard deviation.

**Table 3:** Results of Toxic Element Contamination within Subgroups***.

Element in mcg	Mercury	Lead	Cadmium	Arsenic	Aluminum	Barium	Antimony	Thallium	Tin	Cesium
Allowable limit/day (micrograms)*	2	15	4.1	10	7,000	1,400	2.8	70	200	NE
**Chinese NHPs** (n = 8)										
Average daily exposure (mean)	5.37	4.84	0.160	254	3,760	92.9	0.241	0.102	1.07	0.681
Standard Deviation	14.7	4.79	0.231	713,000	4,580	139	0.423	0.090	1.20	0.860
Highest daily exposure in single sample	41.8	13.0	0.549	2,020	13,000	422	1.93	0.812	8.53	5.45
Average annual exposure	1,960	1,770	58	92,900	1,370,000	33,900	88	37	389	249
Number exceeding daily limit	1	0	0	1	2	0	0	0	0	N/A
Percent with detectable contaminant**	87.5	100	62.5	87.5	87.5	87.5	50	87.5	100	87.5
**Ayurvedic NHPs** (n = 9)										
Average daily exposure (mean)	0.053	4.05	0.0972	0.394	938	68.3	0.196	0.0565	2.45	0.156
Standard Deviation	0.111	6.93	0.114	0.394	1,420	93.2	0.546	0.0898	4.39	0.156
Highest daily exposure in single sample	0.0332	22.3	0.3	1.19	4,290	279	1.65	0.269	13.2	0.0858
Average annual exposure	19	148	35.5	144	342,000	25,000	71.5	20.6	896	57
Number exceeding daily limit	0	1	0	0	0	0	0	0	0	N/A
Percent with detectable contaminant**	44.4	100	55.5	66.7	100	100	88.9	55.5	100	66.7
**Marine-source NHPs**										
(N) tested	9	9	9	9	9	9	5	4	4	4
Average daily exposure (mean)	0.029	7.96	1.67	7.81	1,420	228	0.298	0.194	1.25	0.554
Standard Deviation	0.0497	16.4	2.59	13.6	1,460	220	0.282	0.196	2.270	0.500
Highest daily exposure in single sample	0.0384	51.37	0.272	42.4	1,460	615	0.66	0.0224	4.65	0.951
Average annual exposure	10.6	2,910	611	2,850	518,000	83,300	109	70.9	455	202
Number exceeding daily limit	0	1	0	1	0	0	0	0	0	N/A
Percent with detectable contaminant**	55.5	100	88.9	88.9	100	100	77.8	100	100	100

Category of NHP indicates classification of product in store or company where purchased. This does not necessarily indicate where source materials for the NHPs are initially manufactured or derived.

Average daily exposure represents the mean level after all supplements for each category are incorporated.

* Limits from U.S. California Proposition ^65,^ ^[85]^ and Environmental Protection Agency^[102]^ as per Table 2.

** The limit of detection will vary between analytical laboratories and may thus influence the percent with detectable contaminants when levels are at low concentrations.

*** Tables 3–6 should be interpreted together and in context as there were single outliers (such as the Hg level in one Chinese NHP) that radically skewed the mean and standard deviation.

**Table 4:** Results of Toxic Element Contamination in a Commonly Consumed Prenatal Vitamin Supplement (Results represent average daily exposure at regular dosing).

Element in mcg	Mercury	Lead	Cadmium	Arsenic	Aluminum
Prenatal Allowable limit/day (micrograms)	O.6 for methyl Hg	No level established as acceptable	NE	NE	NE
Regular Dosing: 1 per day					
**Batch**					
A. one single sample	0.002	0.348	negligible	0.444	444
B. Mean –4 samples	0.06	0.44	0.0004	1.65	444
B. SD in lot B	0.0025	0.018	0	0.022	18.3
C. Mean –4 samples	0.031	0.37	0.003	0.76	227
C. SD in lot C	0.0005	0.0038	0	0.016	18.5
D. Mean –4 samples	0.023	0.462	0.004	2.16	485
D. SD in lot D	0.023	0.027	0	0.067	48.4
E. Mean –4 samples	0.077	0.399	0.004	1.38	456
E. SD in lot E	0.0033	0.036	0	0.06	29.4
Highest level in 17 samples	0.08	0.492	negligible	2.23	552
17 sample average (mean)	0.038	0.414	negligible	1.43	380
SD of 17 samples	0.03	0.045	negligible	0.56	144

**Table 5:** Highest Toxicant Levels by Origin of NHP (mcg unless otherwise specified)***.

Mercury			Lead		
Product	Daily dose	Yearly exposure	Product	Daily dose	Yearly exposure
Chinese Herbal	41.9	15,300	Marine	51.4	18,800
Chinese Herbal	0.507	185	Ayurvedic	22.3	8,150
Chinese Herbal	0.397	145	Chinese Herbal	12.8	4,670
Ayurvedic	0.332	121	Chinese Herbal	9.75	3,560
Marine	0.15	54.8	N American	6.54	2,390
N American	0.118	42.9	Chinese Herbal	6.37	2,330
Ayurvedic	0.112	41.1	Marine	6.11	2,230
N American	0.108	39.6	N American	5.05	1,850
Chinese Herbal	0.103	37.8	Chinese Herbal	4.33	1,580
Prenatal	0.080	29.3	N American	3.73	1,360
Chinese Herbal	0.074	27.1	N American	3.32	1,210
Marine	0.052	19.2	Chinese Herbal	3.18	1,160
**Cadmium**			**Arsenic**		
**Product**	**Daily dose**	**Yearly exposure**	**Product**	**Daily dose**	**Yearly exposure**
Marine	6.81	2,490	Chinese Herbal	2020	738,000
N American	4.69	1,710	Marine	42.52	15500
N American	2.01	734	Marine	23.91	8,730
N American	1.86	679	Marine	12.4	4,530
N American	1.02	374	N American	11.16	4,080
N American	1.01	368	Chinese Herbal	6.11	2,230
N American	0.95	347	N American	5.85	2,140
Marine	0.615	225	N American	5.46	2,000
Chinese Herbal	0.549	200	Chinese Herbal	3.91	1,430
Marine	0.539	197	N American	3.62	1,320
Marine	0.514	188	Marine	3.15	1,150
Chinese Herbal	0.505	184	Chinese Herbal	2.9	1,040
**Aluminum**					
**Product**	**Daily dose (mg)**	**Yearly exposure (mg)**			
Chinese Herbal	13.0	4,740			
Chinese Herbal	7.18	2,620			
Chinese Herbal	5.62	2,050			
Ayurvedic	4.29	1,570			
Marine	3.75	1,370			
Marine	3.69	1,350			
Chinese Herbal	2.26	827			
N American	2.07	756			
N American	1.99	728			
Ayurvedic	1.97	720			
Chinese Herbal	1.77	645			
Marine	1.50	548			

Category of NHP indicates classification of product in store or company where purchased. This does not necessarily indicate where source materials for the NHPs are initially manufactured or derived.

*** Tables 3–6 should be interpreted together and in context as there were single outliers (such as the Hg level in one Chinese NHP) that radically skewed the mean and standard deviation.

**Table 6:** Comparison of toxic element contamination of NHPs across published studies.

Study/Year/N	Supplements tested/source	Test Method	Samples containing toxic element (%)					Median concentration (mcg/g) with range	Comments
			Hg*	Cd*	Pb*	As*	Cu*		
Saper et al ^[109]^ 2004, N = 70	Ayurvedic NHPs: In USA grocery stores	XR fluorescence spectroscopy	8.57		18.6	8.57		Mercury: 225 (28–104,000);Lead: 40 (5–37,000);Arsenic: 430 (37–8130)	20% of samples contained toxic elements
Saper et al ^[110]^ 2008, N = 193	Ayurvedic NHPs: USA and Indian manufactured	XR fluorescence spectroscopy	4.1		19.2	27		Mercury: 103.8 (24.5–28200);Lead: 7.5 (2.5–25950);Arsenic: 27.0 (10.5–27.5)	20.7% of samples contained toxic elements; USA manufactured: 21.7% had toxic elements; Indian manufactured: 19.5% had toxic elements
Koh and Woo ^[111]^ 2000, N = 2040	Chinese Proprietary Medicine	Atomic absorption spectroscopy, inductively couple plasma mass spectrometry	1.35		0.38	0.34	0.5	N/A (not available)	Only describes % of samples above the legal allowable limits in ppm or mcg/g: 2.02% of all samples.; Allowable limits defined as:; Mercury 0.5; Lead 20; Arsenic 5; Copper 150
Martena et al ^[48]^ 2010, N = 292	Ayurvedic and Traditional Chinese Medicine	Inductively Coupled Plasma Mass Spectrometry	45		42	36		Mercury: 50 (0.2–171,000);Lead: 13 (0.5–60,000);Arsenic: 7.6 (0.2–89,800)	64% of preparations contained mercury, lead or arsenic; 20% were deemed likely to exceed safety limits
Pakade et al ^[112]^ 2010, N = 14	Ayurvedic Plant source Himalayan	Atomic absorption Spectrophotometry	0.0	35.71	28.57	35.71		Mercury: all below detection limit of 0.02;Lead: (2.5–6);Arsenic: (0.11–.48)	Small study, no mean concentration given
Harris et al ^[113]^ 2011, N = 334	Chinese Herbal Medicines	Inductively Coupled Plasma Mass Spectrometry	42.8		95.8	66.2		Mercury: 0.02 (0.1–0.28);Lead: 0.44 (0.04–8.15);Arsenic: 0.2 (0.08–20)	5% of samples had levels that were of concern.; At least one toxic element detectable in 100% of samples; 34% had detectable levels of all metals; Wild collected plants had higher contamination than cultivated plants
Radhika Singh ^[114]^ 2008, N = 9	Ayurvedic NHPs	Double beam atomic absorption spectrophotometry		100	100	100	100	N/A (not available)	All samples had levels of lead that were 8–80 times the permissible levels; All samples had higher than permissible levels of cadmium; Copper levels were 50–100 times the permissible limit in the samples tested; Arsenic was within permissible levels; Mercury was below detection limit in all samples.

* Hg = Mercury, Cd = Cadmium, Pb = Lead, As = Arsenic, Cu = Copper.

As in Ayurveda, however, heavy metals and metalloids may be intentional components of TCMs. Mercurial compounds by the name of cinnabar (*Zhu Sha* – a type of rock that contains minerals with various elements including mercury sulphide) and calomel (*Qing Fen* – containing mercury chloride), may be prescribed as tranquilizers or for external application, respectively. Calomel, for example, has been used for pediatric teething discomfort, resulting in infant poisoning after application to the gums. Lead (litharge and minium, or *Mi Tuo Seng* and *Qian Dan*) is believed to grant relief from anxiety, convulsions, phlegm and parasites, while arsenic (realgar, or *Xiong Huang*), may be used for treatment of malaria, as well as an antidote to venoms. Copper (chalcanthium, *Dan Fan*) may be used for insomnia. [71].

Various accounts related to TCM contaminated supplement consumption are reported in the literature including arsenic poisoning in a 13 year old girl after ingestion of such supplementation, resulting in pulmonary edema, pericarditis, and eventually renal and liver failure as well as cerebral edema. [35], [65] Chronic lead poisoning has been described in an infant after application of a tongue powder [72] as well as in a woman using a menstrual cramp remedy. [73] Chronic mercury poisoning from TCM preparations has been noted to alter blood pressure and dental health; [74] chronic arsenic exposure has been linked to dermatological lesions and malignancies. [75].

### Sources of Contamination

NHPs pass through multiple stages before landing on store shelves, all of which involve possible routes for toxicant contamination. Raw materials for NHPs often come from international sources, including nations with less stringent controls over water, air and soil pollution [76], [77] and agricultural practices. Plant products [78], [79] may absorb toxic compounds from soil, water and air, [77] while animal products are prone to bioaccumulation in bone and shell materials. [52], [71].

Transport of products creates possible routes for toxicant exposure. Open-bed trucks, for example, may permit transfer of exhaust pollutants into NHP ingredients. [80] Raw materials may be processed in substandard factory conditions allowing contamination, and products may be intentionally diluted with contaminated products or fillers when sold by weight. [72], [81] Finally, intentional additives to supplements may be introduced for perceived therapeutic value.

### Existing Testing & Regulation

Raw materials and bulk ingredients for NHPs may originate from sources located around the world including Asia, Europe, and the Americas. Raw materials are advertised on the internet or displayed at conventions and trade shows in major jurisdictions where they are evaluated and purchased by manufacturing companies. A small number of raw material suppliers feed the many manufacturing establishments. These companies then assemble and package a proprietary formulation of specific products, which are shipped to distributors and retail suppliers for sale. The location of assembly and packaging varies depending on the company.

No testing for safety or contamination is generally required for the sale and distribution of NHPs in many jurisdictions throughout the world. Testing may take place internally by companies wishing to verify identity, strength, composition, quality, and purity; regulatory requirements for such testing, however, are usually nonexistent. In addition, lack of standardization between origin and processing of raw materials results in variation between NHP batches, complicating analysis of efficacy or safety between batches. The sourcing of raw materials for pharmaceuticals may also take place in nations where labor costs are minimal and quality-control less stringent.

In response to pressure from consumers and health professionals, regulatory measures have been established in a few countries, including Canada’s Natural Health Products Regulations (NHPR), established in 2004 by the Natural Health Product Directorate (NHPD). With this initiative, all NHPs require approval by Health Canada for safety, efficacy and quality, and a product license is required for sale within Canada. Receiving such approval can be a very expensive and arduous process for manufacturers. It is unclear what measures are taken by regulators in this country to continually assure the safety, efficacy, purity and quality of each batch of product. In Canada, exemption has been provided to products currently on the market in order to ensure NHP availability while products are being assessed and regulation processes are being put in place. [82].

In America, ‘Guidelines for Good Manufacturing Practices’ (GMP) have been established to promote a system of processes, procedures, and documentation to ensure that NHPs have the composition, quality, and purity they purport to possess. New regulations from the Department of Health and Human Services have been proposed to enable the American Food and Drug Administration to evaluate whether a NHP is reasonably expected to be safe and accurately represented through all phases of preparation for consumer use including manufacturing, packaging and labeling. [83], [84] Clinical trials to assess NHP efficacy are not standard practice in any country, but many observers are calling for regulated research to ensure accuracy of claims prior to market release. Systems have been established in some jurisdictions for reporting of adverse reactions to NHP use.

## Methods

This study was designed to i) determine if toxic element contamination of NHPs and pharmaceuticals is a routine or rare event, and ii) bring attention to the issue of contamination in NHPs and drugs in order to create credible regulatory processes to ensure public safety.

Testing for toxic elements was carried out on a range of pharmaceuticals and over-the-counter NHPs. To the authors’ knowledge, some preliminary work has been done, but no toxic element contamination studies to date have focused on a broad spectrum of NHP preparations available in Canada. The scientific literature was reviewed to explore relevant information regarding NHP contamination. This was done by assessing available scientific literature from Medline, reviewing books and conference proceedings, consulting several toxicologists, and studying various government publications. Searching techniques included key word searches with terms related to NHPs and toxic element contamination.

In this study, undertaken in 2010–2011, 121 commonly used NHPs (as recommended by retailers) were gathered from 8 health-food stores, industry samples, and 3 herbal dispensaries in Ontario and Alberta, Canada. 49 commonly used pharmaceutical medications were also gathered from physician samples and pharmacies in Edmonton, Alberta. In addition, 5 separate batches of one prenatal supplement manufactured in North America and purchased from 5 independent pharmacies in Alberta (with one sample from the first batch, and 4 samples within each of the remaining 4 batches) were tested. This was done to compare toxicant levels between different batches of the same brand, and within samples of the same batch. An effort was made to include NHPs manufactured in differing areas of the world. The country of manufacture may be listed on NHPs, but labels do not provide the source of raw materials used to manufacture final products. Because of this limitation, we were unable to identify products according to the source countries of their components.

The NHPs (excluding the prenatal supplements) were sent for toxic element testing in three separate groups – each group was analyzed at one of three accredited and specialized toxicology laboratories. (ALS Laboratories, CanAlt Laboratories, or Maxxam Analytics). The pharmaceuticals and the prenatal supplements were all tested as one group at ALS laboratories. The full range of element testing was done at ALS laboratories (only toxic element testing was performed at the other labs) but only toxic elements are reported in this study. The results for each group were combined for purposes of analysis. Daily exposure levels were determined for the maximum recommended daily dose for each NHP or drug. When dosing information was based upon volume, the laboratory-determined specific weight of each NHP or drug was factored in, along with the concentration determined by analysis. All laboratories used inductively coupled plasma – mass spectrometry for detection, and the analytical methodology for testing at ALS laboratories (where the majority of products were tested) follows as an example.

Fluid samples were diluted 10-fold with 1.4 M HNO_3_ (SP grade). For solids, 0.1–0.7 g of sample (depending upon available sample size) were subjected to closed-vessel microwave-assisted digestion (MARS-5 oven, 600W. 1 h holding time) using 5 mL concentrated HNO_3_ (SP grade), 0.5 mL H_2_O_2_ (PA grade) and 0.02 ml HF (SP grade). After digestion, solutions were diluted with 1.4 M HNO_3_ (SP grade) providing a final dilution factor of approximately 500. A set of digestion blanks and CRMs were prepared together with each digestion batch. (All solutions were also spiked with 2 µg/L (internal standard) and analyzed by ICP-SFMS (ELEMENT2, Thermoscientific) using a combination of internal standardization and external calibration. Testing for organic pollutants including biotoxins, various synthetic compounds, and various chemical byproducts was not done.

### Reporting of Values

Toxic element contamination results from the laboratories were provided for each NHP and pharmaceutical in ng/g (equivalent to parts per billion), mg/kg (parts per million) or mcg/g (parts per million). While it has been common in the literature to report NHP contamination concentrations, the actual exposure level to individuals was deemed to be of more importance from a clinical and public health perspective. In order to determine how intake levels compare to established limits, calculation of daily intake rather than simple concentration is required. Accordingly, each laboratory result was multiplied by the weight in grams for each NHP and drug tested to ascertain the total amount of contaminant contained per product. This figure was then multiplied by the maximum daily dose recommended in the product instructions for each specific NHP and pharmaceutical in order to determine a maximum daily intake of each product.

While some individuals may consume lower or higher amounts than is recommended for any given NHP or drug, it was determined through discussion with colleagues, patients, pharmacists, NHP distributors and retailers that most people tend to i) consume the maximal recommended NHP dose in order to achieve what is perceived to be the maximum benefit; and ii) take a pharmaceutical dose within the recommended range provided for the product.

### Speciation

Whether an element is toxic or not is determined by many factors including route of exposure, dose, site of accumulation, nutritional status, detoxification biochemistry, and the particular form or species in which the element exists within the body. Different species of elements have the potential to display distinct toxicity patterns. For example, hexavalent chromium (chromium-VI) is highly toxic and carcinogenic while trivalent chromium (chromium-III) is an essential metal involved in lipid and carbohydrate metabolism.

Similarly, inorganic and organic arsenic are both naturally occurring compounds that display different toxicities. While certain inorganic arsenic species are classified as human carcinogens, some forms of organic arsenic, such as arsenobetaine (which accumulates in some aquatic organisms such as shrimp) are relatively nontoxic. Specific forms of some elements also have the potential to be converted within the body to different forms, which changes their properties and potential toxicity. Nonetheless, in this study, only the total amount of each element was determined – no speciation was undertaken to determine the oxidation state or associated organic species.

## Results

Our results indicate varying levels of toxic element contamination in the NHPs and pharmaceuticals tested. Proposed limits of acceptable contamination as determined by various agencies can be found in Table 1– the most commonly used grid, published in California under Proposition 65 [85] is provided within our tables as a reference limit. The overall results of NHP contamination in this study can be found in Tables 2, 3 and 4. Tables 2 and 3 also provide findings within specific subgroups including Ayurvedic, TCM, and marine-source NHPs. Table 5 displays highest toxicant levels in our study by NHP origin; comparison of NHP toxic element contamination across various published studies is provided in Table 6.

Table 3 illustrates that most NHPs tested showed detectable contamination with one or more toxic elements; the number of NHPs exceeding the established daily limit of toxicant exposure for any toxic element, however, was less than 10 percent. These figures reflect single exposures and do not depict total accrued levels resulting from repeated exposures, a noteworthy concern given that some compounds such as lead and cadmium have long half-lives. A wide variation in contamination levels was evident for many toxic elements, frequently associated with the NHP source. Almost all pharmaceuticals also had detectable contamination with multiple toxic elements, but the levels were very low. This may be due, in part, to the fact that most drugs are synthetic, while many NHPs are derived from natural sources. None of the pharmaceuticals had levels which exceeded established limits.

Tables 2 & 3 indicate that several NHPs contained noteworthy concentrations of toxic elements – the degree appears to be linked to the country of manufacture, with higher contamination from mercury, arsenic and aluminum primarily found in products imported from China. Marine-source NHPs averaged the highest level of lead contamination overall. Non-marine NHPs manufactured in North America generally demonstrated the least contamination among samples tested. Although marine-source and Ayurvedic NHPs were most often contaminated, the levels rarely exceeded established toxicity guidelines. It is important that Tables 2–5 are interpreted together and in context as there were single outliers in some NHPs (such as the mercury level in one Chinese NHP), the inclusion of which skewed means and standard deviations.

Table 4 demonstrates that one brand of prenatal supplement was found to have small amounts of lead (mean of 17 samples: 0.414 mcg) in each sample tested. There was consistency of lead concentration within each batch of prenatal supplement analyzed but sizable differences between batches of the same brand. There was wide variation in levels of arsenic between batches of the same-brand prenatal supplement but no levels exceeded the established general daily limit. (Table 1. No specific gestational limit has been defined to the authors’ knowledge.) Table 6 reveals that there are isolated NHPs available on store shelves that appear to be outliers and demonstrate elevated contamination of toxic elements. Several of these products are Chinese herbal NHPs or products which originate from marine sources.

## Discussion

Most of the existing literature on toxic element NHP contamination has reported on contaminant concentrations, with no indication of the dose that an individual would receive at the prescribed rate of intake. In this study, however, we endeavored to estimate daily exposure levels of toxic elements for many NHPs and drugs in an effort to determine if some existing NHPs may pose a health hazard to the consuming public. The results of this study demonstrate that toxic element contamination of NHPs and pharmaceuticals is common, but that none of the drugs and only a few NHPs exceeded established daily limits for contamination when taken on their own. Many people, however, consume multiple different NHPs and/or drugs each day; the total level of toxicant exposure will thus be additive.

The results of our testing on one prenatal supplement brand suggest that ascertaining the safety or purity of one NHP batch does not ensure safety of other same-brand batches. While this finding has significance to all NHPs, gestational exposures merit particular attention as ongoing research continues to link assorted prenatal toxicant exposures and pediatric toxicant levels (including toxic elements) with potentially significant health outcomes. [86], [87].

The findings of this study, however, likely underestimate the overall extent of supplement and pharmaceutical contamination as there are many potential synthetic (e.g. parabens, phthalates, pesticides), biological (e.g. mycotoxins), or petrochemical contaminants not assessed in this research. In the scientific literature, there is a paucity of research reported which explores the spectrum of potential contaminants in NHPs and drugs.

Endeavoring to link specific toxic element exposure levels found in this study directly with health problems is challenging. Causal links between toxic element exposure and illness have, however, been established as extensive evidence from observational studies of exposed populations and individuals, from epidemiological studies of the general population, and from animal studies investigating mechanisms of toxicity has confirmed causality. [88]–[92] Long-term health sequelae of prenatal exposure to toxicants are also documented. [20] Proving simple linearity from exposure to illness, however, is exceedingly difficult because of confounding associated with multiplicity of toxicant exposures and pre-existing body-burdens of contamination. Many individuals now harbor myriad toxicants [21], [22], [93] – compounds with effects that may interact independently, additively or synergistically. [94] Furthermore, the Human Genome Project has confirmed the reality of genetic individuality, establishing the basis for differing propensities for inherent detoxification. [95], [96] The response to toxicants may thus vary from person to person.

It is also of note that the relevance of specific contamination levels found in this study is uncertain. Assigned tolerance limits for toxic element exposures (Table 1) have declined recently, leading some to conclude that no evidence for a safe exposure threshold to toxic elements exists for some compounds. The United Nations, for example, has recently concluded that lead is toxic at very low exposures [97] – a point which is worth mentioning considering the presence of small amounts of lead found in each prenatal sample tested in this study. Furthermore, some elements such as lead and cadmium have prolonged half-lives as they sequester in tissues due to enterohepatic re-circulation and ensuing bioaccumulation. Moreover, the usual standards for established limits are based on animal exposure tolerance which may be superior to human tolerance due to differences in detoxification potential. [98] Accordingly, conclusions on health sequelae from specific levels of exposures are difficult to establish. With evidence of NHP contamination juxtaposed with uncertainty about the clinical and public health significance of these findings, how do we move forward?

Widespread and apparently irreconcilable controversy exists regarding the regulation of NHPs. Many within the medical community have expressed concern about the safety and efficacy of NHPs, [29], [99] while the NHP industry has articulated dismay about the possible introduction of additional regulatory legislation. While some suggest that consumers need protection and that NHPs should receive the same scrutiny as pharmaceutical drugs, [99] NHP advocates often contend that oversight similar to pharmaceutical regulation would be ineffective. To support this contention, they cite published outcomes regarding adverse drug sequelae (ADS) confirming that current pharmaceutical oversight is not working: i) estimated pharmaceutical-related annual mortality in America includes 7,000 deaths related to medication mishaps [41] and 106,000 due to non-error drug effects; [14] and ii) drug-related morbidity is reflected by 2.3 million emergency room visits attributed to ADS annually. [100].

Some propose that NHPs be available only by physician prescription. Others consider this strategy to be ill-advised as most medical doctors have limited toxicological or nutritional training [101] and are often not equipped to evaluate and manage disordered nutritional biochemistry.

A potential solution may involve the NHP industry developing and implementing stringent self-regulatory procedures to ensure safe and reliable NHPs – procedures that are amenable to government oversight by elected officials. ‘Country of Origin’ labeling – including the source country of each component of the product (e.g. ascorbic acid – USA; Vitamin D – New Zealand; folic acid – Japan; etc.) as well as the country where the final product was manufactured, may facilitate full transparency and provide consumers with informed choice. Routine toxicant testing for a wide range of potential contaminants is also required, with full disclosure of toxicant content. The lack of consistency of purity between same-brand batches in this study indicates that ongoing assessment for each batch of every raw material component as well as each batch of manufactured product is needed. This supervised self-regulatory approach is likely more acceptable to industry, and more cost-effective and efficient for governments. Such a process would ensure safety and public confidence.

### Conclusions

NHP use has become commonplace in the 21^st^ century with at least half of the North American and European populations ingesting supplements daily. [23], [30], [31] This study demonstrates, however, that while pharmaceuticals appear to have low concentrations of toxic elements, a small percentage of NHPs have noteworthy concentrations, potentially exposing consumers to adverse health sequelae associated with heavy metal and metalloid bioaccumulation. This is particularly evident in certain NHPs from Chinese herbal sources.

With increasing recognition of widespread iatrogenic illness and potential adverse sequelae resulting from assorted therapies, concerted action is required to secure patient safety and public health in all healthcare domains. [1], [2] Although harm from NHP contamination may be less pressing than literature-documented adverse outcomes associated with pharmaceutical use, [14], [41] toxicant contamination of NHPs appears to be a not-infrequent occurrence. Mechanisms for regulation and monitoring to confirm purity and authenticity in the manufacture of such heretofore unregulated products are therefore necessary. As NHPs are widely consumed and some appear to be indispensable tools in contemporary evidence-based health care, it is imperative to ensure NHP access, quality and safety for the public. Best practices for quality control, developed and implemented by the NHP industry itself with government oversight, is strongly recommended.
